# Symptoms Associated with Victimization in Patients with Schizophrenia and Related Disorders

**DOI:** 10.1371/journal.pone.0058142

**Published:** 2013-03-19

**Authors:** Federico Fortugno, Christina Katsakou, Stephen Bremner, Andrzej Kiejna, Lars Kjellin, Petr Nawka, Jiri Raboch, Thomas Kallert, Stefan Priebe

**Affiliations:** 1 Neurosciences, Mental Health and Sensory Functions Department, School of Medicine and Psychology, Sapienza University, Sant'Andrea Hospital, Rome, Italy; 2 Unit for Social and Community Psychiatry, Barts and the London School of Medicine and Dentistry, Queen Mary University of London, London, United Kingdom; 3 Centre for Primary Care and Public Health, Barts and the London School of Medicine and Dentistry, Queen Mary University of London, London, United Kingdom; 4 Department of Psychiatry, Wroclaw Medical University, Wroclaw, Poland; 5 Psychiatric Research Centre, School of Health and Medical Sciences, Örebro University, Örebro, Sweden; 6 Psychiatric Outpatient Klinik, Dresden, Germany; 7 Psychiatric Department, 1st Medical School, Charles University in Prague, Prague, Czech Republic; 8 Department of Psychiatry, Psychosomatic Medicine and Psychotherapy, Park Hospital Leipzig, and Soteria Hospital Leipzig, Leipzig, Germany; The Nathan Kline Institute, United States of America

## Abstract

**Background:**

Patients with psychoses have an increased risk of becoming victims of violence. Previous studies have suggested that higher symptom levels are associated with a raised risk of becoming a victim of physical violence. There has been, however, no evidence on the type of symptoms that are linked with an increased risk of recent victimization.

**Methods:**

Data was taken from two studies on involuntarily admitted patients, one national study in England and an international one in six other European countries. In the week following admission, trained interviewers asked patients whether they had been victims of physical violence in the year prior to admission, and assessed symptoms on the Brief Psychiatric Rating Scale (BPRS). Only patients with a diagnosis of schizophrenia or related disorders (ICD-10 F20–29) were included in the analysis which was conducted separately for the two samples. Symptom levels assessed on the BPRS subscales were tested as predictors of victimization. Univariable and multivariable logistic regression models were fitted to estimate adjusted odds ratios.

**Results:**

Data from 383 patients in the English sample and 543 patients in the European sample was analysed. Rates of victimization were 37.8% and 28.0% respectively. In multivariable models, the BPRS manic subscale was significantly associated with victimization in both samples.

**Conclusions:**

Higher levels of manic symptoms indicate a raised risk of being a victim of violence in involuntary patients with schizophrenia and related disorders. This might be explained by higher activity levels, impaired judgement or poorer self-control in patients with manic symptoms. Such symptoms should be specifically considered in risk assessments.

## Introduction

Patients with schizophrenia and related disorders have been found to be at higher risk of both being physically violent to others and becoming a victim of physical violence themselves [1]–[10]. In the public perception, the risk that patients may pose to others is more salient than the risk of patients getting harmed themselves. This predominant interest in patients as a potential source of violence is reflected in the scientific literature [11]. In a meta-analysis [1], Choe and colleagues found 30 empirical studies focusing on patients as perpetrators of a crime compared with only 10 studies on patients as victims.

Several studies have demonstrated an association between schizophrenia and a risk of violent behaviour. Based on a meta-analysis of 17 studies [6], Naudts and colleagues suggested that a history of antisocial behaviour and several neurobiological factors were associated with a higher risk of violent behaviour. Nielssen et al (2012) meta-analysis found that a substantial proportion of patients examined after homicide (39%) and assault resulting in serious injury (38%) were in their first episode of psychosis [12]. With respect to psychopathological symptoms, Swanson et al. (2006) reported a higher risk in patients with lower levels of negative symptoms and higher levels of positive symptoms [13]. However, there is a shortage of studies on the symptoms associated with violent behaviour that were conducted in diagnostically homogeneous samples and which have used standardised assessment scales [14]–[17].

There is even less research on the symptoms associated with a risk of patients becoming the victims of violence. There have been suggestions that recent violent victimization is associated with higher levels of symptoms [1], [5], [7], [18], [19].

In particular, positive symptoms with paranoia, and manic symptoms with higher activity levels might be assumed to lead patients into situations with a higher risk of becoming a victim of physical violence. However, to our knowledge, there have been no analyses on what type of symptoms may be specifically linked with a higher or lower risk of becoming a victim of violence. Further limitations within the existing research on victimization of patients with schizophrenia are that studies tend to focus on only one sample each so it may be difficult to generalise the findings beyond the specific characteristics of the sample and the given setting [1].

### Aim

Against this background, we studied what symptoms were associated with becoming a victim of physical violence in patients with schizophrenia and related disorders. Although the focus in this exploratory study was on symptoms associated with victimization, we also addressed what symptoms were associated with patients' reports of being accused of a crime themselves. This was done to obtain a more comprehensive picture of the associations of symptoms with risk that is related to violence and crime. We analysed data from two samples of involuntarily admitted patients with schizophrenia and related disorders. Conducting the analysis in two independent samples was intended to provide findings that are more reliable and less dependent on specific sample and setting characteristics than results obtained in only one sample. Both samples were assessed using the same methods.

## Methods

### Participants

The two independent samples were drawn from two related studies, which assessed psychiatric patients who had been involuntarily admitted to inpatient care. The InvolvE study was conducted in England, whilst the EUNOMIA project studied patients across several European countries [20]. Both studies used a consistent methodology in the recruitment and assessment of patients. They recruited consecutive patients who were involuntarily admitted under the given mental health legislation and between 18 and 65 years of age. The InvolvE study was conducted in 22 hospitals in England. Data for the EUNOMIA study was collected in between 1 and 5 hospitals in 12 countries. Yet, data on victimization was available in only seven countries including the United Kingdom. Some of the EUNOMIA patients in the UK had also been included in the InvolvE, and consequently, the English patients were excluded from the EUNOMIA dataset for this analysis in order to have two independent samples. As a result, the European study sample analysed for the present paper included the patients from six countries, i.e. the Czech Republic, Germany, Lithuania, Poland, Slovakia and Sweden. Details of the rationale, methods and general findings of the two studies have been published elsewhere [21]–[24].

In the English sample, 49.6% of all eligible patients and 74.1% of all patients who were contacted and asked to participate were assessed. In the European sample these figures were 50.0% and 73.8%. From both datasets, we selected all patients with a diagnosis of schizophrenia or a related disorder (ICD-10 F20-F29) [25].

### Instruments

Socio-demographic (age, gender, employment status, housing situation, living situation, frequency of social contacts) and clinical variables (discharge diagnosis, past hospitalization) were obtained from medical records.

Victimization in the year prior to the interview was assessed with a question “in the last year have you been a victim of physical violence?”, which is part of the Manchester Short Assessment for Quality of Life (MANSA) [26] and is answered with ‘yes’ or ‘no’. Being accused of a crime in the year prior to the interview was also assessed with a dichotomised question in the MANSA, i.e. “in the last year have you been accused of crime?”.

Symptoms were assessed on the Extended Version of the Brief Psychiatric Rating Scale [27]. For distinguishing between different types of symptoms, we used the four factor model suggested by Ventura and colleagues [28]. The model has the subscales depression (with the items anxiety, depression, suicidality, guilt), manic symptoms (motor hyperactivity, elevated mood, excitement, distractibility, grandiosity), negative symptoms (blunted affect, motor retardation, emotional withdrawal, self-neglect), and positive symptoms (bizarre behaviour, unusual thought content, disorientation, hallucinations, suspiciousness).

Additionally, reported misuse of non-prescribed drugs or alcohol shortly before admission was assessed in English patients only. Participants' statements on alcohol and substance misuse were dichotomised (yes vs. no). In this sample we also obtained data on the use of coercive measures (i.e. restraint, seclusion, forced medication) during the admission, i.e. after the arrival in the hospital. These details were obtained from medical records, and also dichotomised (at least one coercive measure vs. no coercive measure). Data on substance misuse and the use of coercive measures during the admission procedure was not available in the European sample.

### Procedures

Patients in both studies were involuntarily admitted and assessed within the first week of admission. Assessments were conducted by researchers who had all been trained in administering and rating the BPRS. In the study on the English sample the inter-rater reliability for the BPRS at the end of joint training sessions was 0.90 (Cohen's kappa), and in the European study it was 0.78 (intraclass correlation coefficient). All patients provided written informed consent, and the studies were approved by the relevant national ethics committees [21]–[24].

### Statistical analysis

Univariable logistic regression models were applied to assess the relationship between potential predictor variables and the two outcomes: ‘being a victim of physical violence’ and of ‘being accused of a crime’. Clinical variables included the scores of the BPRS subscales assessing depressive, manic, positive and negative symptoms. Data on previous hospitalization (yes vs. no) was also included. We considered the following socio-demographic variables: gender (male vs. female); age; employment status (unemployed vs. employed/student); housing situation (homeless vs. not homeless); frequency of social contact (one or more per month vs. less frequent contacts); and living situation (living alone vs. living with others). Variables significant at the 10% level were entered into multivariable logistic regression models. In a next step, any variables not significant at the 5% level were removed so that only variables significant at the 5% level remained in the final model. Additionally, we adjusted for country in the models fitted to the EUNOMIA sample. Crude and adjusted odds ratios are reported with 95% confidence intervals and p-values.

Since “being accused of a crime” and “being a victim” were expected to be correlated, we conducted sensitivity analyses to show whether the predictive value of independent variables was specific to each dependent variable. In these analyses, we included the variable “being accused of a crime” in the multivariable models predicting victimization, and the variable “being a victim of physical violence” in the models predicting being accused of a crime.

We conducted two further sensitivity analyses in the English sample by including additional variables in the multivariable logistic regression models. Firstly, the use of coercive measures during the admission was included in the model for predicting victimization, since patients may have referred to these experiences in their reports of having been a victim of physical violence in the last year. Secondly, we included substance misuse in the model predicting being accused of a crime as this has been suggested as an important predictor of violent behaviour in patients with psychosis.

All analyses were conducted in STATA, release 10.1 (Statacorp., College Station, TX, USA).

## Results

### The characteristics of the samples

In English sample 383 patients met the inclusion criteria for this paper. 269 (70.2%) received a diagnosis of schizophrenia (F 20-F 20.9) at discharge. Fifty three (13.8%) patients were diagnosed with acute transient psychotic disorder (F 23) and 43 (11.2%) with schizo-affective disorder (F 25).

In the European sample, 543 patients met the inclusion criteria: Czech Republic = 119; Germany = 65; Lithuania = 78; Poland = 107; Slovakia = 141; Sweden = 33. Two hundred eighty six (52.7%) were diagnosed with schizophrenia at discharge (F 20–F 20.9). A diagnosis of acute transient psychotic disorder (F 23) was given to 86 (15.8%) patients and a diagnosis of schizo-affective disorder (F 25) to 146 (26.9%).

In the English sample 130 patients (33.9%) had experienced at least one coercive measure since the arrival in the hospital, whilst 85/287 (29.6%) patients reported substance misuse prior to the admission.

The socio-demographic and clinical characteristics of the two samples are shown in Table 1.

**Table 1:** Clinical and socio-demographic characteristics of the patients in the English and European samples.

	*English sample*		*European sample*	
	N = 383		N = 543	
**CLINICAL CHARACTERISTICS**				
**Diagnosis (N, %)**	N = 383		N = 543	
F20	269	70.2	286	52.7
F21–29	114	29.8	257	47.3
**Symptoms on BPRS** (mean, SD)	N^a^ = 381		N = 543	
	N^b^ = 379			
Depressive symptoms^a^	2.37	1.16	2.05	0.99
Manic symptoms^b^	2.21	0.89	1.97	0.82
Negative symptoms^a^	1.93	0.88	2.45	1.00
Positive symptoms^b^	3.16	1.21	2.79	1.02
**Past hospitalisation**	N = 375		N = 535	
No	283	75.5	119	22.2
Yes	92	24.5	416	77.8
**Years since first hospitalization**	N = 232		N = 312	
Mean, SD	11.6	8.7	14.5	10.4
**Number of hospitalisations**	N = 192		N = 361	
Mean, SD	3.5	7.0	1.7	3.8
**SOCIO-DEMOGRAPHIC CHARACTERISTICS**				
**Age**	N = 380		N = 543	
Mean, SD	35.9	10.9	40.1	11.8
**Gender** (N, %)	N = 383		N = 543	
Male	275	71.8	267	49.2
Female	108	28.2	276	50.8
**Unemployed** (N, %)	N = 375		N = 542	
No	68	18.1	136	25.1
Yes	307	81.9	406	74.9
**Homeless** (N, %)	N = 378		N = 542	
No	347	91.8	513	94.7
Yes	31	8.2	29	5.4
**Social contacts** (N, %)	N = 341		N = 518	
Less than one per month	128	37.5	153	29.5
One or more per month	213	62.5	365	70.5
**Living situation** (N, %)	N = 370		N = 540	
With others	75	20.3	200	37.0
Alone	295	79.7	340	63.0
**VICTIM/ACCUSED STATUS**				
**Victim of physical violence** (N, %)	N = 357		N = 543	
No	222	62.2	391	72.0
Yes	135	37.8	152	28.0
**Accused of crime** (N, %)	N = 360		N = 542	
No	268	74.4	479	88.4
Yes	92	25.6	63	11.6

### Victim of physical violence/accused of crime status

In the English sample 135/357 patients (37.8%) reported having been a victim of physical violence in the preceding year, while 92/360 (25.6%) stated they had been accused of a crime during the same period of time. Out of 351 responding to both questions, 88 (25.1%) were a victim only, 46 (13.1%) were accused of a crime only, while 45 (12.8%) reported belonging to both categories. 172 patients (49.0%) reported neither experience.

In the European sample 152/543 patients (28.0%) were victims of physical violence in the past year, while 63/542 (11.6%) were accused of crime. One hundred twenty one patients (22.3%) experienced physical victimization only and 32 (5.9%) were accused of crime only. Thirty one (5.7%) patients reported both experiences during past year and 358 (66.1%) stated neither.

Being a victim of physical violence and being accused of a crime were weakly associated (Pearson's r = 0.141, p = 0.008 in the English sample, and r = 0.171, p<0.001 in the European one).

### Associations with being a victim of physical violence

Tables 2 and 3 present the univariable and multivariable associations of patients' clinical and socio-demographic characteristics with reports of having been a victim of physical violence in the last year.

**Table 2:** Univariable and multivariable associations of symptoms and socio-demographic characteristics with being a victim of physical violence in the English sample.

	Victim of		Not victim of							
	physical violence		physical violence		Univariable Regressions			Multivariable Regression^1^		
	N = 135		N = 222		Crude OR	95% CI	*p*-value	Adjusted OR	95% CI	*p*-value
**CLINICAL CHARACTERISTICS**										
**Symptoms on BPRS** (mean, SD)										
Depressive symptoms	2.42	1.12	2.33	1.17	1.07	0.89–1.29	0.479			
Manic symptoms	2.35	0.88	2.10	0.82	1.42	1.10–1.83	0.007	1.39	1.07–1.80	0.012
Negative symptoms	1.90	0.87	1.90	0.86	0.99	0.77–1.27	0.953			
Positive symptoms	3.21	1.20	3.08	1.16	1.09	0.91–1.31	0.334			
**Past hospitalization** (N,%)										
No	103	39.3	159	60.7	1					
Yes	29	32.2	61	67.8	1.36	0.82–2.26	0.232			
**SOCIO-DEMOGRAPHIC**										
**CHARACTERISTICS**										
**Age** (mean, SD)	35.6	10.5	35.7	11.1	1.00	0.98–1.02	0.944			
**Gender** (N, %)										
Male	98	37.7	162	62.3	0.98	0.61–1.59	0.938			
Female	37	38.1	60	61.9	1					
**Unemployed** (N, %)										
No	17	25.8	49	74.2	1					
Yes	115	40.4	170	59.7	1.95	1.07–3.55	0.029	1.88	1.02–3.44	0.042
**Homeless** (N, %)										
No	119	36.6	206	63.4	1					
Yes	15	50.0	15	50.0	1.73	0.82–3.67	0.152			
**Social contacs** (N, %)										
Less than one per month	43	36.4	75	63.6	0.88	0.55–1.41	0.595			
One or more per month	82	39.4	126	60.6	1					
**Living situation (**N, %)										
With others	26	35.6	47	64.4	1					
Alone	105	38.0	171	62.0	1.11	0.65–1.90	0.703			

1 Model fitted to 351 observations.

**Table 3:** : Univariable and multivariable associations of symptoms and socio-demographic characteristics with being a victim of physical violence in the European sample.

	Victim of		Not victim							
	physical violence		of physical violence		Univariable regressions			Multivariable regression^1,2^		
	N = 152		N = 391		Crude OR	95% CI	*p*-value	Adjusted OR	95% CI	*p*-value
**CLINICAL CHARACTERISTICS**										
**Symptoms on BPRS (mean, SD)**										
Depressive symptoms	1.98	0.98	2.08	1.00	0.90	0.74–1.09	0.295			
Manic symptoms	2.16	0.92	1.89	0.76	1.48	1.18–1.85	0.001	1.54	1.21–1.96	<0.001
Negative symptoms	2.28	0.98	2.52	1.00	0.78	0.64–0.95	0.013			
Positive symptoms	2.87	1.02	2.76	1.01	1.12	0.93–1.34	0.229			
**Past hospitalization**										
No	31	26.0	88	74.0	1					
Yes	117	28.1	299	71.9	1.11	0.70–1.76	0.656			
**SOCIO-DEMOGRAPHIC**										
**CHARACTERISTICS**										
**Age** (mean,SD)	40.4	12.6	39.9	11.5	1.00	0.99–1.02	0.670			
**Gender** (N,%)										
Male	70	26.2	197	73.8	0.84	0.58–1.22	0.365			
Female	82	29.7	194	70.3	1					
**Unemployed** (N,%)										
No	33	24.3	103	75.7	1					
Yes	119	29.3	287	70.7	1.29	0.83–2.02	0.258			
**Homeless** (N,%)										
No	143	27.9	370	72.1	1					
Yes	9	31.0	20	69.0	1.16	0.52–2.62	0.713			
**Social Contacts** (N,%)										
Less than one per month	32	20.9	121	79.1	0.61	0.39–0.96	0.033			
One or more per month	110	30.1	255	69.9	1					
**Living situation**										
With others	49	24.5	151	75.5	1					
Alone	102	30.0	238	70.0	1.32	0.89–1.96	0.170			

1 Model fitted to 543 observations; **^2^**Controlling for country.

In the English sample higher levels of manic symptoms were associated with greater odds of becoming a victim of physical violence in the univariable analysis (Odds Ratio (OR) 1.42; 95% CI 1.10–1.83; p = 0.007). This association remained significant in the multivariable analysis (OR 1.39; 95% CI 1.07–1.80; p = 0.012). With regard to socio-demographic characteristics, being unemployed (OR 1.95; 95% CI 1.07–3.55; p = 0.029) was significantly associated with increased odds of becoming a victim of violence in both univariable and multivariable models (OR 1.88; 95% CI 1.02–3.44; p = 0.042).

In univariable analyses carried out in the European sample higher levels of manic symptoms on the BPRS subscale (OR 1.48; 95% CI 1.18–1.85; p = 0.001) were associated with greater odds of being a victim of physical violence. Higher levels of negative symptoms (OR 0.78; 95% CI 0.64–0.95; p = 0.013) were associated with lower odds of victimization. Only the association with manic symptoms remained significant (OR 1.54; 95% CI 1.21–1.96; p<0.001) in the multivariable model. In univariable analysis, as compared to those with one or more social contacts per month, those with less frequent contacts had a significantly reduced odds of being a victim (OR 0.61; 95% CI 0.39–0.96; p = 0.033). This association did not reach statistical significance in the multivariable model. No other socio-demographic variables were associated with being a victim of physical violence in univariable or multivariable analyses in the European sample.

### Associations with being accused of a crime

Associations of symptoms and patient characteristics with being accused of a crime are shown in Tables 4 and 5.

**Table 4:** : Univariable and multivariable associations of symptoms and socio-demographic characteristics with being accused of a crime in the English sample.

	Accused of crime		Not accused of crime		Univariable regressions			Multivariable regression^1^		
	N = 92		N = 268		Crude OR	95% CI	*p*-value	Adjusted OR	95% CI	*p*-value
**CLINICAL CHARACTERISTICS**										
**Symptoms on BPRS** (mean, SD)										
Depressive symptoms	2.42	1.16	2.37	1.17	1.03	0.85–1.26	0.748			
Manic symptoms	2.24	0.86	2.20	0.90	1.05	0.80–1.37	0.724			
Negative symptoms	1.92	0.98	1.90	0.84	1.02	0.78–1.33	0.893			
Positive symptoms	3.16	1.21	3.16	1.19	1.00	0.82–1.22	0.972			
**Past Hospitalization** (N, %)										
No	64	26.4	23	73.6	1					
Yes	67	25.0	201	75.0	0.93	0.53–1.61	0.789			
**SOCIO-DEMOGRAPHIC**										
**CHARACTERISTICS**										
**Age** (Mean, SD)	33.2	10.0	36.8	11.15	0.97	0.95–0.99	0.008	0.97	0.95–0.99	0.015
**Gender** (N, %)										
Male	80	31.0	178	69.0	3.37	1.75–6.51	<0.001	2.92	1.44–5.89	0.003
Female	12	11.8	90	88.2	1			1		
**Unemployed** (N, %)										
No	6	9.4	58	90.6	1			1		
Yes	85	29.2	206	70.8	3.99	1.66–9.59	0.002	4.48	1.82–11.04	0.001
**Homeless** (N, %)										
No	78	23.8	250	76.2	1					
Yes	14	46.7	16	53.3	2.80	1.31–6.00	0.008			
**Social Contacts** (N, %)										
Less than one per month	37	29.8	87	70.2	1.36	0.83–2.25	0.226			
One or more per month	49	23.8	157	76.2	1					
**Living situation** (N, %)										
With others	9	12.2	65	87.8	1					
Alone	80	28.7	199	71.3	2.90	1.38–6.11	0.005			

1 Model fitted to 352 observations.

**Table 5:** : Univariable and multivariable associations of symptoms and socio-demographic characteristics with being accused of a crime in the European sample.

	Accused of crime		Not accused of crime		Univariable regressions			Multivariable regression^1,2^		
	N = 63		N = 479		Crude OR	95% CI	*p*-value	Adjusted OR	95% CI	*p*-value
**CLINICAL CHARACTERISTICS**										
**Symptoms on BPRS (mean, SD)**										
Depressive symptoms	2.08	1.01	2.04	0.99	1.04	0.80–1.35	0.776			
Manic symptoms	2.24	0.79	1.93	0.82	1.50	1.12–2.00	0.006	1.46	1.08–1.97	0.015
Negative symptoms	2.18	0.98	2.48	0.99	0.73	0.55–0.96	0.027			
Positive symptoms	2.79	0.90	2.78	1.03	1.01	0.78–1.30	0.956			
**Past hospitalisation** (N/%)										
No	10	8.5	108	91.5	1					
Yes	52	12.5	364	87.5	1.54	0.76–3.14	0.231			
**SOCIO-DEMOGRAPHIC**										
**CHARACTERISTICS**										
**Age** (mean, SD)	37.1	11.4	40.5	11.8	0.98	0.95–1.00	0.034			
**Gender** (N/%)										
Male	44	16.5	223	83.5	2.66	1.51–4.69	0.001	2.82	1.57–5.08	0.001
Female	19	6.9	256	93.1	1			1		
**Unemployed** (N/%)										
No	12	8.8	124	91.2	1					
Yes	51	12.6	354	87.4	1.49	0.77–2.88	0.238			
**Homeless** (N/%)										
No	59	11.5	453	88.5	1					
Yes	4	13.8	25	86.2	1.23	0.41–3.65	0.711			
**Social Contacts** (N/%)										
Less than one per month	12	7.9	140	92.1	0.55	0.29–1.07	0.079			
One or more per month	49	13.4	316	86.6	1					
**Living situation** (N/%)										
With others	20	10.0	180	90.0	1					
Alone	42	12.4	297	87.6	1.27	0.72–2.24	0.402			

1 Model fitted to 543 observations; **^2^**Controlling for country.

In the sample of English patients there were no statistically significant associations between BPRS symptoms and being accused of crime in either univariable or multivariable models. With respect to socio-demographic characteristics, male gender (OR 3.37; 95% CI 1.75–6.51; p<0.001), younger age (OR 0.97; 95% CI 0.95–0.99; p = 0.008), being unemployed (OR 3.99; 95% CI 1.66–9.59; p = 0.002), being homeless (OR 2.80; 95% CI 1.31–6.00; p = 0.008), and living alone (OR 2.90; 95% CI 1.38–6.11; p = 0.005) were associated with greater odds of being accused of a crime in univariable analyses. Only the associations with male gender (OR 2.92; 95% CI 1.44–5.89; p = 0.003), younger age (OR 0.97; 95% CI 0.95–0.99; p = 0.015), and being unemployed (OR 4.48; 95% CI 1.82–11.04; p = 0.001) remained significant in multivariable models.

In the European sample higher levels of manic symptoms (OR 1.50; 95% CI 1.12–2.00; p = 0.006) were associated with increased odds of being accused of a crime in univariable analyses. Higher levels of negative symptoms (OR 0.73; 95% CI 0.55–0.96; p = 0.027) were associated with reduced odds of reporting the same experience. Only the association with manic symptoms remained significant in the multivariable analysis (OR 1.46; 95% CI 1.08–1.97; p = 0.015). In this sample, male gender was positively associated with being accused of a crime in both univariable (OR 2.66; 95% CI 1.51–4.69; p = 0.001) and multivariable analyses (OR 2.82; 95% CI 1.57–5.08; p = 0.001). Younger age was associated with greater odds of being accused of a crime in the univariable analysis only (OR 0.98; 95% CI 0.95–1.00; p = 0.034).

### Sensitivity analyses

When we included reports of having been accused of a crime in the multivariable model with being a victim of violence as a dependent variable, the associations with symptoms were virtually unchanged in both samples. The variables significant in the multivariable models for being accused of a crime as a dependent variable retained the significance of their associations when recent victimization was included in the same models.

In the English sample we conducted further sensitivity analyses by including the experience of coercive measures during the admission as a variable in the prediction of victimization; and substance misuse prior to admission as a variable in the prediction of being accused of a crime.

The experience of coercive measures during admission was not significantly associated with victimization, and did not alter the associations with manic symptoms and unemployment in the multivariable models.

Self-reported substance misuse was associated with being accused of a crime in univariable (crude OR = 3.99, 95% CI 2.21 – 7.20, p<0.001) and multivariable analyses (adjusted OR 2.99; 95% CI 1.59 – 5.63; p = 0.001). When this variable was included in the multivariable model, it did not alter the associations with socio-demographic variables.

## Discussion

### Main findings

In two large independent samples of involuntarily admitted patients with schizophrenia and related disorders, higher levels of manic symptoms were associated with increased odds of reporting having been a victim of physical violence in the previous year. These associations held true in multivariable analyses that were adjusted for the influence of other influential variables. In these multivariable models manic symptoms remained the only type of symptoms that was significantly linked with reports of experiencing physical violence in the previous year. Manic symptoms therefore indicate a higher risk of having been victimised in involuntary patients with schizophrenia and related disorders. Higher levels of such symptoms were also associated with reports of having been accused of a crime in one of the two samples.

### Strengths and limitations

To our knowledge, this is the first study investigating what types of symptoms are particularly associated with the risk of becoming a victim of violence in patients with schizophrenia and related disorders. We analysed data from two independent samples to permit replication and reduce the risk of having findings that are context and setting specific. Although the samples showed significant differences in a number of characteristics, the findings on the association between manic symptoms and victimization were consistent. The sizes of both samples provided sufficient power for the statistical analysis to detect effects of a clinically relevant size. All participants were patients involuntarily admitted to hospital. Whilst it remains to be addressed in further studies to what extent the findings can be generalised to other groups of patients with schizophrenia, involuntarily admitted patients represent a group with a particularly high risk of being involved in violence (risk of physical harm to themselves or others is a common reason for involuntary admission) and the clinical challenge to predict risk in this group is especially relevant. The relatively high level of victimization, i.e. 37.8% in one sample and 28.0% in the other, should have increased the sensitivity of the analyses as compared to other samples (e.g. voluntary inpatients) with lower levels of victimization. Both samples were studied using similar methods which helped to compare the findings, and psychopathological symptoms were assessed on a standardised scale by well-trained researchers who had achieved good inter-rater agreements [21]–[24]. A further strength of the study is that symptoms were assessed within the first week after admission when symptom levels were still high and probably reflected the type of symptoms patients had prior to their admission, rather than at a later stage when symptoms are supposed to have changed as a result of treatment. In the English sample we showed that the findings are not influenced by the experience of coercive measures, in the case of patients reporting victimization during the admission procedure, or by substance misuse as self-reported by patients, in the case of patients being accused of a crime.

The study has several limitations. Most importantly, victimization was assessed only through patient self-reports and these may be influenced by memory and reporting bias. People with manic symptoms may be more likely to report experiences of physical violence or interpret minor incidents as physical violence. Moreover, the experience was assessed using only one question. The analysis did therefore not distinguish between different types of violence in terms of severity, duration, or the number and kind of people involved. The same limitations apply to the assessment of having been accused of a crime. The questions on victimization and being accused of a crime used different terms and are therefore not equivalent. This may have led to an underestimation of the association between being a victim and a perpetrator of violence. The association between current symptoms and victimization and being accused of a crime were established retrospectively rather than prospectively. This may have influenced the findings although one can only speculate as to whether the experience of physical violence might have caused later manic symptoms.

The two samples were selective as not all eligible patients who had been involuntarily admitted and who fulfilled the inclusion criteria within the study periods were interviewed. The recruitment of involuntarily admitted patients to research studies is challenging and both studies had included about 50% of the eligible patients which has been described as good for this type of research [29]. Any selection bias may have influenced the absolute levels of reported victimization, but should be less problematic for establishing predictive associations, which are supposed to be more robust against selection bias. The sensitivity analyses considering coercive measures during admission and self- reported substance misuse as potential confounders was possible in only one of the two samples, and substance misuse was assessed using only one self- reported variable.

Finally, the association between manic symptoms and victimization could have been due to unobserved confounders, and potentially mediating factors, such as levels of outdoor activities, were not assessed.

### Comparison with literature

Involuntary inpatients are admitted because they may pose a risk to themselves or others. Thus, high rates of victimization and being accused of a crime may be expected in such populations. The levels of victimization in the samples studied here are in line with other findings in the literature [1], [5]. The percentages of those being accused of a crime were also consistent with other research. Previous studies on the perpetration of violence reported rates ranging from 14.2% among voluntary inpatients one month before hospitalization to 50.4% in committed inpatients up to four months before hospitalization [2].

Whilst it has been previously suggested that higher symptom levels in general may be linked to victimization [30]–[35], this is the first study to provide evidence on the specific importance of manic symptoms. This association may have several explanations. Patients with higher levels of manic symptoms such as hyperactivity and elevated mood are more likely to be active, spend time outdoors and have social contacts. This can increase the likelihood of getting into situations that involve physical violence. Grandiosity, distractibility and excitement may impair patients' judgement in social situations, and lead to inappropriate assessments of the intention of others and their willingness to accept the wishes of the patient. Patients may misread the significance of other people's communication and take more risks which can increase the likelihood to engage in situations that escalate into violence. Manic symptoms can also reduce the self-control of patients so that they may trigger situations in which they become the victims of violence. Patients with manic symptoms can also be more irritable and dysphoric which can lead to aggression although one would assume that this would be linked to being accused of a crime as much as being a victim of violence, and we have found this association in one of the samples. These explanations are neither mutually exclusive nor exhaustive.

Several previous studies reported an association between symptom levels of persecutory delusions, suspiciousness, hallucinations, and grandiosity with a higher risk of committing a crime [13], [15], [16], [36], [37]. These associations however have not been consistent, and some studies report no significant associations between symptoms and criminal behaviour. There may be a tendency for studies with lower prevalence of criminal behaviour to find associations between symptoms and criminal behaviour, whilst these cannot be shown in samples with higher risk levels. Accordingly, we identified an association between manic symptoms and being accused of a crime in the European sample, which had a lower percentage of patients who were reported to have been accused of a crime. It was not found in the English sample in which relatively more patients stated to have been accused of a crime.

With respect to socio-demographic characteristics as potential predictor variables of victimization, being unemployed was linked with a higher risk of victimisation in one of the two samples. This may be due to two factors. The regulated environment at most workplaces may limit the risk of becoming a victim of physical violence, and patients in employment are likely to have a generally better social functioning and therefore be able to avoid situations with a risk of violence.

Male gender was linked with a higher probability of being accused of a crime in multivariable analyses in both samples, while being unemployed and younger age in only one sample. All these associations are in line with what has been previously reported in the literature [14], [38]–[42].

## Conclusions

The findings suggest that manic symptoms indicate a higher risk of becoming a victim of physical violence. Such symptoms should be considered in risk assessments and addressed in treatment plans for reducing the risk of victimization.

Future research should explore the mediating factors that put patients with more severe manic symptoms at a higher risk of victimization, and develop and test specific treatment strategies to reduce the risk for such patients.
